# Does Formal Complexity Reflect Cognitive Complexity? Investigating Aspects of the Chomsky Hierarchy in an Artificial Language Learning Study

**DOI:** 10.1371/journal.pone.0123059

**Published:** 2015-04-17

**Authors:** Birgit Öttl, Gerhard Jäger, Barbara Kaup

**Affiliations:** 1 Department of Psychology, Eberhard Karls University, Tübingen, Germany; 2 Department of Linguistics, Eberhard Karls University, Tübingen, Germany; Northeastern University, UNITED STATES

## Abstract

This study investigated whether formal complexity, as described by the Chomsky Hierarchy, corresponds to cognitive complexity during language learning. According to the Chomsky Hierarchy, nested dependencies (context-free) are less complex than cross-serial dependencies (mildly context-sensitive). In two artificial grammar learning (AGL) experiments participants were presented with a language containing either nested or cross-serial dependencies. A learning effect for both types of dependencies could be observed, but no difference between dependency types emerged. These behavioral findings do not seem to reflect complexity differences as described in the Chomsky Hierarchy. This study extends previous findings in demonstrating learning effects for nested and cross-serial dependencies with more natural stimulus materials in a classical AGL paradigm after only one hour of exposure. The current findings can be taken as a starting point for further exploring the degree to which the Chomsky Hierarchy reflects cognitive processes.

## Introduction

### Formal language theory and the Chomsky hierarchy

It has been a very fruitful guiding hypothesis of linguistic research since the mid-twentieth century that all natural languages are—despite their superficial diversity—fundamentally similar. While this general hypothesis is still controversial (see [1] for a skeptical view), it has led to many profound insights especially in the domain of grammar. To facilitate the study of the common core of natural language grammars with mathematical precision, Noam Chomsky (see for instance [2], [3]) proposed a number of idealizations, such as:

- A natural language is considered as an infinite set of well-formed sentences, each of which is a finite string of words.
- Whether or not a string of words is a grammatical sentence does not depend on its meaning.
- The distinction between grammatical sentences and ungrammatical strings is categorical.

Every set of finite strings of symbols is a *formal language*. According to Chomsky, a comprehensive theory of syntax has to identify among the formal languages the *possible human languages*, i.e. the class of string sets that could be acquired as native language by a human infant. Chomsky [4] furthermore devised a classification of the formal languages into a nested hierarchy of complexity classes, the so-called *Chomsky Hierarchy* (a more comprehensive discussion of the Chomsky Hierarchy in relation to Artificial Grammar Learning can be found in [5]). The least restrictive—and therefore most complex—class are the *Recursively enumerable* or *Type 0* languages. These are all formal languages for which there is an algorithm enumerating all its elements. The more restrictive classes are the *context-sensitive (Type 1)* languages, the *context-free (Type 2)* languages, and the *regular (Type 3)* languages (the names *context-sensitive* and *context-free* are purely historically motivated and should not be taken at face value). It is uncontroversial among linguists that virtually all well-studied natural languages require at least context-free (the question whether this holds for all natural languages is currently hotly disputed; see [6], [7] and the references cited there) and not more than context-sensitive complexity. Whether or not all natural languages are context-free proved to be a fairly intricate problem which was only solved in 1984, when Huybregts [8] demonstrated that Swiss German is not context-free. Even Swiss German—and other natural languages that have non-context-free features—is much less complex than the most complex context-sensitive languages. Only a slight extension of the complexity of context-free languages is sufficient to cover all natural languages. Joshi and colleagues [9] proposed to refine the Chomsky Hierarchy by the additional level of *mildly context-sensitive languages* that include all context-free languages and are a proper sub-class of the context-sensitive languages. Based on current knowledge, all natural languages are mildly context-sensitive.

The three levels of the Chomsky Hierarchy that are relevant for the study of natural languages—regular, context-free and mildly context-sensitive languages—are characterized by the admissible *dependencies* within strings that they admit. Two positions within a string *s* that belongs to a language *L* are dependent if altering the symbol at the first position (such as deleting or replacing it, or adding additional material before or after) requires a concomitant change at the other position to preserve membership in L. To illustrate this with a simple example, consider the following English sentences:

1.
  - It *either* rains *or* snows.
  - It *neither* rains *nor* snows.

Sentence (1a) is a grammatical sentence of English. If we replace *either* by *neither*, we also have to replace *or* by *nor* to preserve grammaticality. Therefore, there is a dependency between *either* and *or*.

Now let us consider a more complex pattern:

1.
  - The cat_1_ runs_1_.
  - The cat_1_ that the dogs_2_ know_2_ runs_1_.
  - The cat_1_ that the dogs_2_ that the lady_3_ owns_3_ know_2_ runs_1_.
  - The cat_1_ that the dogs_2_ that the lady_3_ that … owns_3_ know_2_ runs_1_.

**Fig 1:**
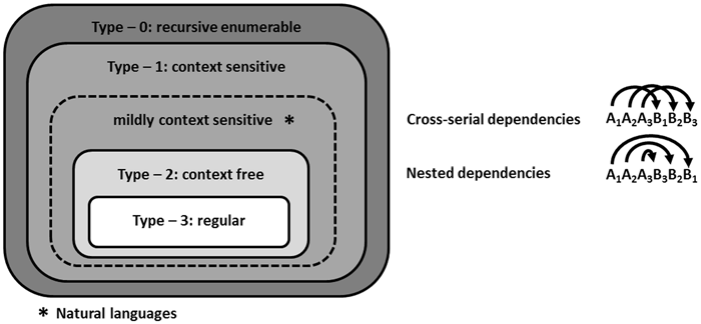
The Chomsky Hierarchy including mildly context-sensitive languages.

In English, the subject noun and the verb of a clause must agree in number—i.e. there is a dependency between the two positions—regardless of the number of words occurring between them. Such dependencies are called *unbounded*. In particular, we may insert an embedded clause between the two positions which contains its own subject and verb. This operation can be applied recursively, leading to an arbitrarily high number of nested dependencies (as far as the grammar of English is concerned, that is; the sentences quickly become incomprehensible due to processing constraints). Context-free languages, but not regular languages, may contain an unbounded number of nested dependencies. So the pattern above demonstrates English not to be regular. While context-free languages may contain an unbounded number of *nested dependencies*, they never contain an unbounded number of *cross-serial dependencies*. Mildly context-sensitive, but not context-free languages may contain this type of unbounded crossing dependencies. Fig 1 shows an example string for nested and cross-serial dependencies and their location in the refined Chomsky Hierarchy.

As discussed above, the Chomsky Hierarchy classifies formal languages according to some quite abstract notion of complexity. It is far from obvious whether this notion of complexity corresponds to some empirically testable notion of cognitive complexity. Still, it has been hypothesized in the literature (most influentially in [10], [11]) that languages higher up in the hierarchy are harder to process—for humans as well as for other species—than those at the bottom of the hierarchy. This dovetails nicely with results from formal language theory regarding the processing complexity of these language classes. Time complexity of the recognition problem for regular languages is linear in length of the input string, while space complexity is constant [12]. In contradistinction, standard parsing algorithms for context-free languages (such as the CYK-algorithm) require cubic time complexity (meaning: the number of steps that a deterministic computer requires to decide whether a given string belongs to a given context-free grammar is bounded by a cubic function of the length of the string) and quadratic space complexity (meaning: the maximal number of memory cells is bounded by a quadratic function of the length of the string) [13]. Mildly context-free languages have a still higher processing complexity in this sense. Standard parsing algorithms for mildly context-sensitive languages (for Tree Adjoining Languages, to be precise [14]; the notion of “mild context-sensitivity” is sometimes also applied to a family of slightly more powerful language classes) such as the CYK-algorithm have a time complexity of O (*n* ^6^) and a space complexity of O (*n* ^4^) (meaning: the number of computing steps is bounded by a polynomial function of 6^th^ degree of the length *n* of the string, and the number of memory cells by a polynomial function of 4^th^ degree) [13]. This suggests the hypothesis that the cognitive processing complexity for humans of mildly context-sensitive languages is still higher than the complexity of context-free languages.

It should be added that the hypothesized correspondence between formal and cognitive processing complexity is, at best, suggestive. Leaving aside the obvious differences between deterministic Turing machines and the human brain, the mentioned results apply to general-purpose algorithms, i.e. algorithms that are capable of recognizing all regular/context-free/mildly context-sensitive languages. If human processing of sentences or of strings of some artificial languages employs more specialized strategies applicable only to sub-classes thereof, the mentioned complexity results do not necessarily carry over. Also, the complexity of the grammar induction process is orthogonal to the issue of processing complexity, and formal language theory has little to say about this. With these qualifications in mind, one possibility is to consider the Chomsky Hierarchy as a heuristics for processing complexity. This leads to the hypothesis that for humans, mildly context-sensitive languages are harder to process than context-free languages (which are in turn harder to process than regular languages). If so, cross-serial dependencies corresponding to the mildly-context sensitive complexity level in the Chomsky Hierarchy should be harder to process than nested dependencies and dependencies of the complexity level regular; nested dependencies should in turn be harder to process than less complex dependencies of the complexity level regular within the Chomsky Hierarchy. This hypothesis builds on the *Derivational Theory of Complexity* [15] according to which memory load and thus processing difficulty rises with increasing syntactic complexity [16]. However, it should be noted that in this theory syntactic complexity was defined as the number of transformations necessary to arrive at the deep structure of a sentence, and thus differed from the notion of syntactic complexity as defined by the Chomsky Hierarchy.

### Artificial grammar learning

The hypothesis that more complex languages are harder to process than less complex languages of course presupposes that human participants are in principle able to process cross-serial dependencies (and all less complex dependencies) even in languages that lack meaning or prosody, such as artificial languages. Studying syntactic dependencies in artificial languages has the advantage that syntactic processing can be studied in a very controlled and structured manner. In contrast to natural languages, language components such as semantics or prosody can be excluded from the cognitive process. Thus, syntactic processing can be studied in its pure form. Additionally, prior language knowledge which might vary between participants does not pose a problem.

An experimental paradigm that is widely used to study the cognitive processing of syntactic dependencies is the *artificial grammar learning* (AGL) paradigm [17]. In an AGL experiment, participants are presented with sequences of symbols following a particular rule. Here the idea is that symbols correspond to words, sequences correspond to sentences and the underlying rule corresponds to the syntactic dependencies between words in a sentence. Importantly, participants are blind to the rule underlying the sequences at the beginning of the experiment. After the first half of the experiment, participants are typically informed about the existence of the rule, and are asked to judge whether or not the subsequently presented sequences follow the rule from the first half of the experiment.

We will now turn to the results of studies investigating artificial grammar learning with languages of different complexity levels. Empirical evidence suggests that participants can easily process dependencies of the complexity type regular in artificial languages (i.e. [10], [11]). For nested dependencies, the empirical evidence is less clear because in studies using artificial languages it has been proven difficult to disentangle whether participants processed nested dependencies, as claimed by [10] and [11], or rather applied particular strategies not involving the processing of nested dependencies ([18], [19], [20]). More recent studies however demonstrated that participants are able to process nested and also cross-serial dependencies in artificial languages, at least under very restrictive conditions ([21], [22]) (we will come back to this point at the end of the introduction). It can be concluded that participants indeed seem to be able to process syntactic dependencies up to the complexity level mildly context-sensitive, in the absence of semantics, at least under very controlled and restricted conditions. Thus, the presupposition for investigating differences with respect to processing syntactic dependencies can be seen as satisfied.

Empirical studies assessing the difference in processing of nested and less complex regular dependencies provided evidence incorporable with the view that the Chomsky Hierarchy can be considered a heuristics for processing complexity ([11], [23], [24], for a review see [25]). With respect to the complexity difference between nested and cross-serial dependencies, Chesi and Moro [26] however argue against the idea of the Chomsky Hierarchy as an adequate reflection of cognitive language processes. Based on the *Syntax Prediction Locality Theory* (SPLT, [27]), the authors propose that sentences with nested dependencies should be harder to process than sentences with cross-serial dependencies due to a higher memory load for nested dependencies compared to cross-serial dependencies [26]. Thus, the SPLT claims that the cognitive load involved in processing nested and cross-serial dependencies is not the same. More specifically, according to the SPLT [27], when processing dependencies in a sentence, memory cost increases with increasing distance. That means that for example in Fig 1, the memory cost for the dependency between A_1_ and B_1_ in the nested sequence is higher (distance: four intervening elements) than the memory cost for the same dependency in the cross-serial sequence (distance: two intervening elements). When further comparing the memory costs for nested and cross-serial dependencies it becomes evident that for nested dependencies, distances between dependent elements differ between dependencies, whereas for cross-serial dependencies distances between dependent elements are the same for all dependencies. Because memory costs increase with increasing distance between dependent elements and long distance dependencies carry the most weight, nested dependencies are predicted to lead to higher memory costs compared to cross-serial dependencies. According to the SPLT this predicts more difficulties when processing nested dependencies compared to cross-serial dependencies (please refer to [27] for a detailed derivation). Thus, [27] and [26] suggest the opposite of what the Chomsky Hierarchy would predict. In line with this view, Bach and colleagues [28] showed that natural language sentences with nested dependencies are judged as less comprehensible compared to sentences with cross-serial dependencies.

Additional evidence for this view comes from two studies that investigated the processing of syntactic dependencies in artificial languages. Uddén and colleagues [21] investigated the cognitive processing involved in cross-serial and nested dependencies. Participants were presented with visual sequences of letters containing either nested dependencies (context-free) or cross-serial dependencies (mildly context-sensitive) over a period of nine days. Letter sequences were embedded into a larger sequence of irrelevant letters to make the target sequences less obvious. Learning effects were observed for both types of dependencies, suggesting that humans are able to process dependencies of the mildly context-sensitive complexity level in an AGL paradigm when language learning lasts over several days. A processing advantage for cross-serial over nested dependencies also became evident in this study, consistent with [26] and the SPLT [27] and contrary to what would be predicted by the Chomsky Hierarchy. However, it is important to note that the stimulus set employed in this study was fairly small. In addition, the stimuli were visual and not presented sequentially in this study. Thus, in order to further investigate the compatibility of the Chomsky Hierarchy with cognitive processes in an AGL setting it would certainly be beneficial to apply more natural learning conditions, such as a large stimulus set, auditory stimuli and a sequential presentation style. This was partly realized in a recent study by de Vries and colleagues [22]. Participants were presented with auditory stimuli in sequential order. A combination of a serial reaction time (SRT) task and an AGL paradigm was applied. In addition, language exposure time was much shorter (approximately half an hour) as compared to Uddén and colleagues [21]. The results of this study were in line with the earlier findings in showing easier processing for cross-serial dependencies compared to nested dependencies. This study thus provides further evidence that cognitive processes for nested and cross-serial dependencies do not follow the predictions of the Chomsky Hierarchy when syntactic processing is investigated in an artificial language and thus independent of other language components. However, even though conditions in this study were closer to natural conditions, the applied set of stimuli was still fairly small. Thus, findings by [21] and [22] suggest that under very restricted experimental conditions participants are able to process nested and cross-serial dependencies and show higher performance for cross-serial compared to nested dependencies.

The present study aims at investigating whether the learning effect for both dependency types can be generalized to an experimental setting that is closer to natural conditions, in particular concerning the set size of the stimulus material. In addition, the present study investigates whether under these conditions, performance for nested dependencies is better than that for cross-serial dependencies (as predicted by the Chomsky Hierarchy), or whether the differences are the other way around (as predicted by SPLT). As in the study by de Vries and colleagues [22] auditory stimuli were presented sequentially to the participants and language exposure time was rather short. An AGL paradigm was applied comparable to the study by Uddén and colleagues [21]. By converging the experimental settings of Uddén and colleagues [21] and de Vries and colleagues [22], the present study allows investigating learning effects without extensive language exposure and when participants are confronted with language material that is closer to natural language. In line with findings from de Vries and colleagues [22] and Uddén and colleagues [21] we expect to find learning effects for both nested and cross-serial dependencies, but with better performance for the cross-serial compared to nested dependencies.

## Experiment 1

### Method

#### Participants

Thirty participants took part in the experiment (age: *M*(*SD*) = 26.87 (4.02) years; gender: 24 female; native language German: 29). All participants were right handed and received course credit or a financial reimbursement of 8 Euro per hour for participating in the study. Participants were randomly assigned to the nested dependency group or the cross-serial dependency group (15 participants per group).

#### Ethics statement

The experimental testing was in agreement with the guidelines for good scientific practice at the University of Tübingen (Germany). This was checked and approved by the Head of Psychology, Faculty of Science, University of Tübingen. He functioned as an independent individual judge who was in no way involved in the study. Prior to the experiment participants were informed that they were free to terminate the experiment at any time without facing disadvantages. After participants signed an informed consent form, a number was assigned to each participant. This number was associated with the recorded data of each participant throughout the whole experiment and data analysis. Thus, participants' anonymity was always preserved; at no point could the recorded data be associated with a participant's name.

#### Apparatus and Stimuli

Spoken auditory stimuli consisted of 20 syllable pairs in which one syllable belonged to category A and one syllable to category B, see Table 1. As in the study by Friederici and colleagues [11], category membership was indicated by the vowel of each syllable (category A: ‘e’, ‘i’, category B: ‘o’, ‘u’). Element pairing was signified by the identical first letter of both syllables within a pair. In the example syllable pair ‘del—dol’, ‘del’ belongs to category A and ‘dol’ to category B. The first letter ‘d’ indicates that both syllables belong to the same element pair. All syllables were spoken by a female native German speaker and were recorded with Audacity (syllable length *M*(*range*) = 689.95 ms (476 ms–906 ms)). Syllables were merged to sequences of either 4 (short), 6 (medium) or 8 (long) syllables using Matlab (R2011b, 32-bit win). As a result of this, there was no rising or falling intonation across the whole sequence. All syllables were counterbalanced across syllable position and number of occurrence for the Category A elements (first half of sequence). Category B elements (second half of sequence) were separately re-arranged for each sequence length according to either nested dependencies (reverse order of A elements) or cross-serial dependencies (same order as A elements). As a consequence, there were minor differences for the nested dependencies with respect to how often a syllable occurred in each position in the B part of the sequences. All sequences were presented only once throughout the entire experiment in one random order to all participants. During the learning phase, participants were presented with 240 sequences (80 sequences each for short, medium, long) following one language (containing either nested dependencies or cross-serial dependencies). In the test phase, participants were then presented with 360 sequences, of which 180 sequences (60 sequences for short, medium, long) belonged to the dependency type in the learning phase (correct trials) and 180 sequences (60 sequences each for short, medium, long) did not belong to the dependency type in the learning phase (incorrect trials). The sequences presented in incorrect trials always followed the language that was not presented in the learning phase. The experiment was programed in Matlab (R2010a, 32-bit maci) using the Psychophysics Toolbox (Version 3.0.8). Participants performed the experiment on a MacBook Pro and were presented with the auditory stimuli via headphones. A short questionnaire was completed after the experiment to obtain information about potential strategy usage.

**Table 1:** Stimulus material of Experiment 1.

Class A	Class B
del	dol
sted	stod
bem	bom
jelz	jolz
pfes	pfos
schip	schop
riw	row
fid	fod
hiz	hoz
zib	zob
lef	luf
sek	suk
kem	kum
pegs	pugs
wel	wul
tix	tux
miv	muv
nist	nust
xim	xum
gid	gud

#### Procedure

Throughout the entire experiment, a white fixation cross appeared on a grey background while a sequence was played. After every twentieth trial, participants had the option to take a short break. In the learning phase, a sequence could be initiated by pressing the space bar, and participants were instructed to listen to the syllable sequences. Importantly, they were not informed about the existence of a rule underlying the sequences. In 25% of the trials, they were asked to repeat the last heard sequence in order to assess their state of alertness. The test phase followed immediately after the learning phase. Participants were informed that all sequences from the previous phase followed an underlying rule. They were further informed that they would now be presented with sequences either consistent with the rule from the learning phase or not. They were instructed to judge for each sequence whether it followed the rule, by pressing the c-key for “yes” and the m-key for “no”. Participants could take as much time as needed to indicate their decision by button press. The experiment lasted approximately one hour.

### Results

All reported analyses were performed in Matlab (R2011b, 32-bit win) and SPSS (version 20). D prime (*d’*) as a measure for performance was calculated for each dependency type. In order to ensure that none of the four classes hits, misses, false alarms and correct rejections equaled zero, 0.5 was added to each class for all participants [29]. A *d’* of 0 corresponds to chance level (50%). Greenhouse-Geisser correction for sphericity violation in repeated measures ANOVAs was applied when appropriate. In case of a significant finding, effect size was calculated using Cohen’s d (*d*) for *t*-tests and partial eta square (*η*_*p*_^*2*^ *)* for ANOVAs.

To evaluate the performance of the participants, a *t*-test against zero was calculated for each dependency type. In both groups, participants performed significantly above chance (nested: *t*(14) = 4.45, *p* = .001, *d* = 1.15; cross-serial: *t*(14) = 5.55, *p* <.001, *d* = 1.43). Thus, learning effects were present for both types of dependency. There was no difference between the two nested and cross-serial dependencies with respect to performance as indicated by the results of a *t*-test for independent samples, (*t*(28) = 0.08; *p* >.90).

To assess potential performance differences between the two dependencies at earlier time points in the test phase, we divided the test phase into 3 blocks (Block 1: trial 1–120, Block 2: trial 121–240, Block 3: trial 241–360) and calculated *d’* for each block separately. A 2-x-3 ANOVA with the factors dependency (nested, cross-serial) and block (1, 2, 3) revealed a significant main effect of block (*F*(1.30,36.46) = 5.51, *p* <.05, *η*_*p*_^*2*^ = 0.16), but no interaction (*F* <1) and no main effect of dependency (*F* <1). Follow-up 2-x-2 ANOVAs showed a significant improvement from Block 1 to Block 2 (*F*(1,28) = 9.57, *p* <.01, *η*_*p*_^*2*^ = 0.26) and from Block 1 to Block 3 (*F*(1,28) = 4.51, *p* <.05, *η*_*p*_^*2*^ = 0.14) but not from Block 2 to Block 3 (*F* <1).

To investigate performance for sequences of different lengths, *d’* was calculated for each sequence length separately. A 2-x-3 ANOVA with the factors dependency (nested, cross-serial) and sequence length (short, medium, long) showed a significant main effect of sequence length (*F*(1.41,39.45) = 27.88, *p* <.001, *η*_*p*_^*2*^ = 0.50) but no main effect of language (*F*<1) and no interaction (*F*(1.41,39.45) = 2.07, *p* = .15). Separate 2-x-2 ANOVAs with the factors dependency and sequence length indicated that performance was better for short sequences as compared to medium (*F*(1,28) = 43.74, *p* <.001, *η*_*p*_^*2*^ = 0.61) and long sequences (*F*(1,28) = 26.83, *p* <.001, *η*_*p*_^*2*^ = 0.49). No difference in performance was observed for medium and long sequences (*F* <1).

### Discussion

Results showed that for both types of dependencies, a learning effect was observed but performance did not differ between nested and cross-serial dependencies. Throughout the test phase performance seemed to improve for both types of dependency in a similar way. This suggests that learning effects for nested and cross-serial dependencies in an AGL paradigm are also present under more natural conditions (larger stimulus set, spoken auditory stimuli, sequential presentation style). Furthermore, as in previous studies, no evidence could be obtained for the idea that the Chomsky Hierarchy reflects cognitive processes, at least not with regard to the proposed complexity difference for nested and cross-serial dependencies.

However, our results are not fully consistent with these earlier findings by de Vries and colleagues [22] and Uddén and colleagues [21] in showing no better performance for cross-serial as compared to the nested dependencies. Participants in our study showed a higher learning performance for short compared to medium or long sequences, which is consistent with the results of previous studies showing that processing difficulty rises for materials with more than two dependencies ([28], [22]).

Interestingly, two participants in the nested dependencies group reported that they rated the sequences based on the first element pair (i.e.: nested dependencies: **A**_**1**_A_2_A_3_B_3_B_2_ **B**_**1**_; cross-serial dependencies: **A**_**1**_A_2_A_3_ **B**_**1**_B_2_B_3_). As incorrect sequences in the test phase were sequences from the respective other dependency, sequences necessarily differed in their arrangement of the element pairs. Hence, by paying attention to only one element pair and ignoring the rest of the sequence, it was possible to respond correctly in the test phase without having learnt the underlying language. Importantly, this alternative strategy would not reflect language processing on a context-free or mildly context-sensitive complexity level but rather the acquisition of a dependency type of the less complex regular complexity level. Attending only to the first element pair will be referred to as *first-element-pair-strategy* in what follows. Tracking only the last element pair while ignoring the rest of the sequence will be referred to as *last-element-pair-strategy*. It can be concluded that the application of one of these strategies might have affected learning performance in this experiment and possibly overshadowed performance differences between the two dependency types. Therefore, Experiment 2 was conducted to assess the degree to which learning effects from Experiment 1 can be explained by these strategies. The learning phase was kept identical to Experiment 1. To control for the application of alternative strategies, incorrect sequences in the test phase were adapted.

## Experiment 2

### Method

#### Participants

Forty-four participants took part in the experiment. Four participants had to be excluded from the analyses because it could not be guaranteed that they were blind with respect to the underlying rule at the beginning of the experiment. These four participants were most likely not blind to the underlying rule because they already had taken part in an AGL study in our laboratory in the past (two participants), reported after the experiment that they had been told by a friend that the goal of the learning phase was to find a rule prior to the experiment (one participant), completed the experiment while the instruction sheet for the experimenter (including the goal to find the rule in the learning phase) was accidently left in the cabin with the participant (one participant). Out of the 40 remaining participants (age: *M*(*SD*) = 21.78(3.83) years; gender: 32 female; native language German: 40) eight participants were left handed. Participants received course credit or a financial reimbursement of 8 Euro per hour. As in Experiment 1, participants were randomly assigned to one of the two learning groups (20 participants per group).

#### Ethics statement

The experimental testing was in agreement with the guidelines for good scientific practice at the University of Tübingen (Germany). This was checked and approved by the Head of Psychology, Faculty of Science, University of Tübingen. He functioned as an independent individual judge who was in no way involved in the study. Prior to the experiment participants were informed that they were free to terminate the experiment at any time without facing disadvantages. After participants signed an informed consent form, a number was assigned to each participant. This number was associated with the recorded data of each participant throughout the whole experiment and data analysis. Thus, participants' anonymity was always preserved; at no point could the recorded data be associated with a participant's name.

#### Apparatus and Stimuli

The stimulus material was identical to the stimulus material from Experiment 1 except for the syllable sequences in the test phase. In the test phase of this experiment, participants were only presented with sequences of medium length (n = 240). Half of the trials were sequences consistent with the dependency type from the previous learning phase (correct trials) and the other half of trials were inconsistent with the dependency type from the learning phase (incorrect trials) (see Table 2). One third of the incorrect trials were sequences following the dependency type not presented in the learning phase (Subset 1; similar to Experiment 1). In order to control for the first-element-pair-strategy, one third of the incorrect trials were sequences violating the dependency type from the learning phase (target rule) while preserving the first element pair (Subset 2; see 3rd and 4th incorrect trial of nested dependency and cross-serial dependency in Table 2). Thus, participants only tracking the first element pair would judge this sequence as correct even though it does not follow the target rule. To control for the last-element-pair-strategy, the remaining third of the incorrect trials violated the target rule while preserving the last element pair in the sequence (Subset 3; see 5th and 6th incorrect trial of nested dependencies and cross-serial dependencies in Table 2). Thus, participants who are only paying attention to the third (last) element pair would incorrectly judge these sequences as correct.

**Table 2:** Correct and incorrect trials in Experiment 2.

Nested dependencies	Cross-serial dependencies	Accuracy	Subset and Strategy
A_1_A_2_A_3_B_3_B_2_B_1_	A_1_A_2_A_3_B_1_B_2_B_3_	Correct	
A_1_ **A**_**2**_A_3_B_1_ **B**_**2**_B_3_	A_1_ **A**_**2**_A_3_B_3_ **B**_**2**_B_1_	Incorrect	Subset 1: Similar to Experiment 1
A_3_ **A**_**2**_A_1_B_3_ **B**_**2**_B_1_	A_3_ **A**_**2**_A_1_B_1_ **B**_**2**_B_3_	Incorrect	Subset 1: Similar to Experiment 1
**A**_**1**_A_2_A_3_B_2_B_3_ **B**_**1**_	**A**_**1**_A_2_A_3_ **B**_**1**_B_3_B_2_	Incorrect	Subset 2: First-element-pair-strategy
**A**_**1**_A_3_A_2_B_3_B_2_ **B**_**1**_	**A**_**1**_A_3_A_2_ **B**_**1**_B_2_B_3_	Incorrect	Subset 2: First-element-pair-strategy
A_1_A_2_ **A**_**3**_ **B**_**3**_B_1_B_2_	A_1_A_2_ **A**_**3**_B_2_B_1_ **B**_**3**_	Incorrect	Subset 3: Last-element-pair-strategy
A_2_A_1_ **A**_**3**_ **B**_**3**_B_2_B_1_	A_2_A_1_ **A**_**3**_B_1_B_2_ **B**_**3**_	Incorrect	Subset 3: Last-element-pair-strategy

*Note*. For incorrect trials element pairs consistent with the respective language are bold faced.

#### Procedure

The procedure was identical to the procedure in Experiment 1 with the only difference being that each participant was now presented with a different random order of the stimuli.

### Results

Experiment 2 investigated the learnability of nested dependencies and cross-serial dependencies while controlling for the first- and the last-element-pair-strategy. Data analysis was performed as in Experiment 1.

**Fig 2:**
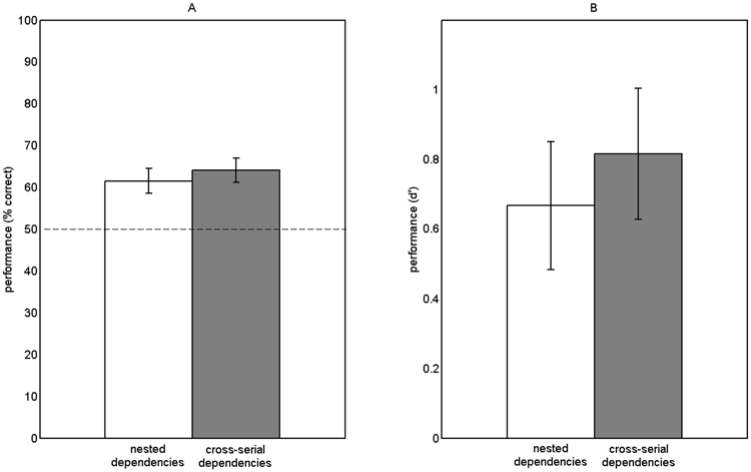
Results from Experiment 2. A: Mean percentage correct *(SE)* for nested and cross-serial dependencies, respectively. B: Mean *d’s (SE)* for nested and cross-serial dependencies, respectively.

Fig 2 presents the overall performance for each group in percentage correct along with the corresponding *d’*. Results showed that participants performed significantly above chance for both types of dependencies (nested: *t*(19) = 3.65, *p* <.01, *d* = 0.82; cross-serial: *t*(19) = 4.33, *p <*.001, *d* = 0.97). Thus, as in Experiment 1, a learning effect was observed for both languages. Similar to Experiment 1, no differences in performance between dependency types was observed (*t*(38) = -0.56; *p* = .58) leading to the conclusion that both dependencies were learned equally well.

In order to control for the use of the first- and the last-element-strategy, *d’* was calculated with an error rate taking only sequences for the first- or the last-element-strategy into account (‘*d’*_*first*_’, ‘*d’*_*last*_’). The idea was that participants applying one of these strategies would rate incorrect sequences that preserve the particular element pair falsely as correct. Thus, taking only these strategy-specific sequences into account, a higher error rate and hence a smaller *d’* should be observed for strategic response behavior. In other words, a low *d’*_*first*_ and a low *d’*_*last*_ indicate that participants applied the respective strategy, whereas a high *d’*_*first*_ and a high *d’*_*last*_ show high performance independent of strategic behavior. For both dependency types, *d’*_*first*_ was significantly above chance (nested: *t*(19) = 3.06, *p* <.01, *d* = 0.68; cross-serial: *t*(19) = 4.35, *p* <.001, *d* = 0.97) as well as *d’*_*last*_ (nested: *t*(19) = 3.37, *p* <.01, *d* = 0.75; cross-serial: *t*(19) = 3.34, *p* <.01, *d* = 0.75). Thus, participants showed learning effects for both types of dependencies independent of the first-element-pair-strategy or the last-element-pair-strategy.

Whether or not participants applied the first-element-pair-strategy or the last-element-pair-strategy was additionally assessed on an individual participants’ analysis. Here we compared the number of yes- to the number of no-responses in each subset of the incorrect sequences. If participants did not learn the underlying dependency, they should press ‘yes’ as often as ‘no’ in Subset 2 and Subset 3 of incorrect sequences. On the other hand, if participants acquired the underlying type of dependency, they should press ‘no’ more often than ‘yes’ in Subset 2 and Subset 3 of incorrect stimuli. In contrast, if participants applied a strategy they should press ‘yes’ more often than ‘no’ in Subset 2 and Subset 3 of the incorrect stimuli. To assess whether participants generally tended to press ‘yes’ more often than ‘no’ independent of any rule knowledge or strategy usage, yes/no- responses were also analyzed in Subset 1 of the incorrect stimuli. In this subset, sequences neither followed the first-element-pair-strategy nor the last-element-pair-strategy. Three binomial tests with a critical value of 0.5 (corresponding to chance) were calculated for each participant separately (first-element-pair strategy, last-element-pair strategy, general tendency to say ‘yes’). Out of all participants, three participants in the nested dependency group responded significantly more often with ‘yes’ in one of the two strategy subsets (Subset 2 and Subset 3), but importantly did not respond significantly more often with ‘yes’ in the general tendency subset (Subset 1). When these three participants were left out of the main analysis, learning performance within and between groups was in line with the results reported above. We therefore consider it safe to conclude that the observed learning effects do not reflect the use of the first- or the last-element-pair strategies.

As in Experiment 1, performance was assessed throughout the test phase at three time points (Block 1: trial 1–80, Block 2: trial 81–160, Block 3: trial 161–240). A 2-x-3 ANOVA with the factors dependency type (nested, cross-serial) and block (1, 2, 3) revealed no significant main effect of block (*F*(1.58,60.13) = 2.31, *p* = .12), no significant main effect of dependency type (*F* <1) and no significant interaction (*F*(1.58,60.13) = 2.45, *p* = .11). Thus, performance for both types of dependency did not differ significantly across the test phase.

### Discussion

Results showed that when controlling for the first-element-pair and the last-element-pair-strategy, participants still showed a learning effect for both types of dependency. Thus, Experiment 2 replicated the finding from Experiment 1 while controlling for strategic behavior.

## General Discussion

This study investigated whether the Chomsky Hierarchy is reflected in cognitive learning processes when participants are confronted with new (artificial) language material. Nested dependencies (context-free) and cross-serial dependencies (mildly context-sensitive) were investigated. According to the Chomsky Hierarchy, cross-serial dependencies are located at a higher level of complexity than nested dependencies, which should lead to lower learning performance for cross-serial compared to nested dependencies in a cognitive learning task. In contrast, based on [28], the SPLT [27] would predict a higher memory load when processing nested compared to cross-serial dependencies in natural language. Therefore, according to the SPLT, processing nested dependencies should result in more difficulties than processing cross-serial dependencies in natural language, a prediction that is also made by Chesi and Moro [26]. Furthermore, de Vries and colleagues [30] proposed that nested dependencies involving a reversed copying process should be more difficult to learn than the cross-serial dependencies involving a simple copying process in artificial languages. Empirical studies provided evidence for this hypothesis in showing better learning performance for cross-serial as compared to nested dependencies in AGL experiments after extensive language exposure [21] and a processing benefit for cross-serial over the nested dependencies in an SRT-AGL experiment after approximately half an hour [22]. Adding onto these findings, the current study aimed at investigating the cognitive adequacy of the Chomsky Hierarchy by combining the approaches by de Vries and colleagues [22] and Uddén and colleagues [21]. In an AGL paradigm [21] spoken auditory stimuli were presented in a sequential presentation style [22]. Language exposure was rather short consistent with de Vries and colleagues [22]. Importantly, more natural stimulus conditions were realized by constructing a larger set of stimuli. We hypothesized that participants would acquire both types of dependencies in our experimental setup. In line with Uddén and colleagues [21] and de Vries and colleagues [22] we expected to find learning effects for both dependency types but better performance for the cross-serial than for nested dependencies.

Results showed a learning effect for both types of dependencies, but no difference between nested and cross-serial dependencies in Experiment 1. In Experiment 1, we could not be sure that participants did indeed learn the underlying dependency without applying the first-element-pair-strategy and/or the last-element-pair-strategy. Therefore, Experiment 2 was conducted, controlling for the use of alternative strategies. Results from Experiment 2 were in line with findings from Experiment 1, suggesting that nested dependencies, reflecting the context-free complexity level, and cross-serial dependencies, reflecting the mildly context-sensitive complexity level, are learnable in principle with our artificial language material. These results therefore extent existing findings [21] and [22] by demonstrating that participants are able to acquire nested and cross-serial dependencies within only one hour of exposure in a larger set of artificial language stimuli. Beyond this, however, our results do not match the predictions that can be derived from the Chomsky Hierarchy, and are in this respect consistent with the findings by Uddén and colleagues [21] and de Vries and colleagues [22].

The absence of a significant performance difference between nested and cross-serial dependencies in our study is however also not in line with predictions by the SPLT [27] predicting better performance for cross-serial compared to nested dependencies for natural language processing. This might suggest that predictions by the SPLT for language comprehension [27] are not easily transferable to the processing of artificial languages where syntactic processing is studied in isolation. Findings from [21] and [22] that investigated the processing of nested and cross-serial dependencies in artificial languages however speak against this conclusion since their findings are incorporable with predictions by the SPLT.

One explanation for why performance for cross-serial dependencies did not differ from the performance for nested dependencies in our study could be that nested dependencies might have had a processing advantage relative to cross-serial dependencies. More specifically, it is possible that processing the innermost dependency of nested dependency sequences (e.g. ‘A_3_ B_3_’ in the sequence A_1_ A_2_ A_3_ B_3_ B_2_ B_1_) was particularly easy for participants in our study because no element intervened the dependent elements at this position. It would be interesting to investigate whether this potential processing advantage for nested dependencies could be demolished by adding an intervening element at the innermost position for both dependency types, i.e. a “dummy” syllable (‘D’) (we thank an anonymous reviewer for this suggestion). This would lead to sequences such as ‘A_1_ A_2_ A_3_ D B_3_ B_2_ B_1_’ for nested dependencies and sequences such as ‘A_1_ A_2_ A_3_ D B_1_ B_2_ B_3_’ for cross-serial dependencies. According to the Chomsky Hierarchy adding a dummy variable would not affect predictions with respect to nested and cross-serial dependencies. Also the SPLT would not predict that adding a dummy variable would affect the memory load for nested dependencies in a different way than the memory load for cross-serial dependencies. In order to draw definite conclusions this empirical question would need to be addressed in a future study. If adding a dummy variable would lead to a higher performance for cross-serial compared to nested dependencies one would however have to incorporate findings by Uddén and colleagues [21], who found higher performance for cross-serial than for nested dependencies without having an intervening dummy variable.

In this context it is interesting to note that there is one difference between our study and that of Uddén and colleagues [21] which could be made responsible for the different results, namely language experience [30]. The study by Uddén and colleagues [21] investigated language learning in Dutch participants. Since Dutch, as Swiss German, contains grammatical constructions with cross-serial dependencies, speakers of Dutch are experienced in processing this type of dependencies [22]. The obtained processing advantage for cross-serial dependencies over nested dependencies in the study by Uddén and colleagues [21] could therefore be attributed to the fact that Dutch participants were investigated [30]. De Vries and colleagues [22] compared the processing of nested and cross-serial dependencies in Dutch and German participants, as speakers of German, in contrast to Dutch speakers, should be more experienced in processing nested dependencies. In contrast to the predictions, their findings did not speak for a strong influence of language experience. Still, it could be the case that in the current study, language experience compensated the general processing advantage of cross-serial dependencies, because participants were native speakers of German (with the exception of one participant in Experiment 1). This hypothesis is strengthened by findings from Rohrmeier and colleagues [31]. In this study, language experience affected learning performance in an AGL experiment when acquiring dependencies of the context-free complexity level. Also Gervain and colleagues [32] showed that language experience affected performance of participants in an AGL experiment. To what degree language experience plays a role in language processing is an interesting question in the context of language learning and should be investigated further in future research.

In addition, it is of course possible that the differences with respect to learning performance appeared due to the different language material or the different control task in the learning phase. In our study, participants had to repeat aloud the last sequence in 25% of the trials in the learning phase to ensure that they were paying attention to the stimuli. Anecdotally, some participants reported after the end of the experiment that they found this task very difficult. Therefore, it could be the case that participants in the learning phase developed coping mechanisms in order to perform well in the control task of the learning phase. These coping mechanisms could have been beneficial with respect to extracting a rule in the test phase and thus might have contributed to the absence of performance differences between dependencies. Irrespective of this, the present study extends previous findings ([21], [22]) in that it shows that language learning can take place in a classical AGL setting within as little time as one hour and in a more natural language environment involving a larger stimulus set and the sequential presentation of spoken auditory stimuli.

Furthermore, we would like to note that a growing body of research suggests that a rule based system similar to the one discussed here for syntax also underlies phonology (for a review, see [33]). For studies employing the artificial grammar paradigm, in which an artificial language is being taught in the absence of semantic information, it would then be difficult to tell whether what is being investigated is syntax or phonology. In any case, considerations concerning complexity differences between the different types of dependencies investigated in the current study should hold independent of whether these dependencies are phonological or syntactic in nature. However, future studies are clearly needed that try to disentangle phonology from syntax in the artificial grammar paradigm. Investigations comparing the learning of the different rule based systems would certainly constitute an important step towards a better understanding of the cognitive processes involved in language processing.

Finally, in the present study, we only investigated two types of dependencies, and did so under restricted learning conditions. Future studies could take this study as a starting point for further exploring cognitive processing of complex dependencies in artificial languages and comparing it with predictions from the Chomsky Hierarchy and memory models such as the SPLT. The limit of learnability with respect to language complexity in the Chomsky Hierarchy would be particularly interesting. Thus, by assessing the learnability of dependencies located higher than cross-serial dependencies in the Chomsky Hierarchy, cognitive processing limits could be identified and could be compared with predictions by the SPLT. This would shed further light onto the compatibility of cognitive and formal complexity.

## Conclusion

Findings of the current study demonstrate the learnability of nested dependencies (context-free) and cross-serial dependencies (mildly context-sensitive) in a more natural AGL setting (spoken auditory stimuli, sequential presentation, larger stimulus set) after only one hour of language exposure. Differences between the dependency types did not become evident. Thus, the current finding suggests that formal complexity and cognitive complexity are not two sides of the same coin. Therefore, this study provides further empirical evidence for the conclusion recently drawn by Chesi and Moro [26] that the Chomsky Hierarchy does not reflect cognitive processes. Cognitive processing limits might become evident when investigating formally more complex dependencies.
